# Cervical dystonia and pain: characteristics and treatment patterns from CD PROBE (Cervical Dystonia Patient Registry for Observation of OnabotulinumtoxinA Efficacy)

**DOI:** 10.1007/s00415-014-7343-6

**Published:** 2014-04-22

**Authors:** P. David Charles, Charles H. Adler, Mark Stacy, Cynthia Comella, Joseph Jankovic, Aubrey Manack Adams, Marc Schwartz, Mitchell F. Brin

**Affiliations:** 1 Department of Neurology, Vanderbilt University Medical Center, 1161 21st Ave S, Suite A-1106 MCN, Nashville, TN 37272 USA; 2 Department of Neurology, Mayo Clinic, Scottsdale, AZ USA; 3 Department of Neurology, Duke University Medical Center, Durham, NC USA; 4 Department of Neurological Sciences, Rush-Presbyterian-St. Luke’s Medical Center, Chicago, IL USA; 5 Department of Neurology, Baylor College of Medicine, Houston, TX USA; 6 Allergan, Inc., Irvine, CA USA; 7 MedNet Solutions, Inc., Minnetonka, MN USA; 8 School of Medicine, University of California, Irvine, CA USA

**Keywords:** Botulinum toxin, Cervical dystonia, Dystonia, Pain

## Abstract

To compare profiles of subjects with and without cervical dystonia (CD)-associated pain, to evaluate the contribution of pain and the motor component of CD on quality of life, and to compare the initial botulinum toxin treatment paradigm between pain groups, baseline data were used from the CD Patient Registry for Observation of OnabotulinumtoxinA Efficacy (CD PROBE), a multicenter, prospective, observational registry designed to capture real-world practices and outcomes for onabotulinumtoxinA CD treatment. Subjects were divided into no/mild pain [Pain Numeric Rating Scale (PNRS) score 0–3] and moderate/severe pain groups (PNRS score 4–10). Descriptive and differential statistics were utilized to compare groups. 1,037 subjects completed the first treatment session, reported baseline botulinum toxin status, and completed baseline PNRS. Those with no/mild pain were significantly older at baseline. Those subjects with moderate/severe pain had higher Toronto Western Spasmodic Torticollis Rating Scale Severity (17.7 ± 5.1 vs. 16.2 ± 5.6, *p* < 0.0001) and Disability (12.7 ± 6.1 vs. 7.5 ± 5.6, *p* < 0.0001). CD subjects with moderate/severe pain received a higher mean dose (177.3 ± 82.9 vs. 158.0 ± 67.1 U, *p* = 0.0001) of onabotulinumtoxinA and were injected in more muscles (4.1 ± 1.4 vs. 3.7 ± 1.2, *p* < 0.0001) at initial treatment. CD PROBE clearly demonstrates the frequency of pain in CD and substantiates its importance when determining an optimal treatment paradigm. Future analyses of CD PROBE will further our understanding of the treatment patterns and outcomes related to onabotulinumtoxinA therapy for this disabling condition.

**Electronic supplementary material:**

The online version of this article (doi:10.1007/s00415-014-7343-6) contains supplementary material, which is available to authorized users.

## Introduction

Cervical dystonia (CD) represents the most common form of adult onset focal dystonia, and pain is one of its most prevalent and disabling features [1–3]. Idiopathic CD typically presents in midlife with insidious onset, and is a neurological disorder with sustained involuntary neck muscular contraction resulting in twisting and turning movements and abnormal head and shoulder postures [3–6]. Because oral medications rarely provide adequate symptomatic relief without intolerable side effects, botulinum toxin (BoNT) injection is widely regarded as first-line therapy for CD [7]. For those who are either no longer adequately responding to BoNT injection, surgical interventions, including selective peripheral denervation or deep brain stimulation, may be considered [8].

The CD Patient Registry for Observation of OnabotulinumtoxinA Efficacy (CD PROBE) is the largest observational study of subjects with CD. The main objectives are to generate data to improve understanding of the demographic and clinical presentation of those suffering from CD, as well as to define the effectiveness and safety profile for onabotulinumtoxinA treatment [9]. Herein, we focus on analyses related to pain, a highly debilitating feature associated with the condition. The role of pain in CD pathophysiology and severity is not well understood. Thus, study analyses compare the demographic and clinical profiles between those with no/mild and moderate/severe CD-associated pain, evaluate the contributions of pain and the motor component of CD on quality of life, and compare the initial onabotulinumtoxinA treatment paradigm between groups.

## Methods

Cervical Dystonia Patient Registry for Observation of OnabotulinumtoxinA Efficacy is a prospective, multicenter, observational registry that enrolled subjects with CD from January 12, 2009 to August 31, 2012 at 88 sites in the United States. Since the aim was to describe the utilization of onabotulinumtoxinA within this rare disease, the study size was determined as the number of subjects who could be reasonably recruited within this time frame. A comprehensive description of the methods of CD PROBE has been previously published [9].

### Subjects

Briefly, subjects with a physician’s diagnosis of CD were either naïve to BoNT therapy, new to the physician’s practice, or had not received BoNT for ≥16 weeks if a previous participant in a clinical trial. Subjects could be enrolled if they met any of these inclusion criteria, which were designed to exclude subjects who are on a stable and optimized botulinum toxin therapy, as these subjects may not show a great change from their condition at study baseline. Exclusion criteria involved planning elective surgery during the study period; pregnancy, nursing, or planning a pregnancy; a history of non-compliance with medical treatment; or any condition or situation that, in investigator opinion, could place the subject at risk, confound the registry data, or interfere significantly with subject participation in the registry.

### Study assessments

For this analysis, subject-reported measures included the Pain Numeric Rating Scale (PNRS), a validated, single-item question on the current level of pain (range 0–10) [10–12], with established cut-points of 0–3 for mild, 4–6 for moderate, and 7–10 as severe [13, 14], and the CD Impact Profile-58 (CDIP-58), a validated questionnaire comprised of eight subscales (Head and Neck Symptoms, Pain and Discomfort, Upper Limb Activities, Walking, Sleep, Annoyance, Mood, and Psychosocial Functioning, each ranging from 0 to 100) [15]. Subjects also completed a work productivity questionnaire developed for this registry [9]. Physician assessments included severity of CD (mild, moderate, or severe, compared to the most severe CD case seen or imagined), classification of the predominant subtype (anterocollis, laterocollis, retrocollis, or torticollis), and the Toronto Western Spasmodic Torticollis Rating Scale (TWSTRS), a CD-specific questionnaire composed of subscales for Pain (range 0–20), Severity (range 0–35), and Disability (range 0–30) [16]. The onabotulinumtoxinA dose and the number of muscles injected at first treatment were also evaluated.

### Registration, protocol approvals, and subject consents

This study is registered with ClinicalTrials.gov (NCT00836017). Each participating center obtained institutional review board approval, and written informed consent was obtained from each subject prior to any study procedures being performed.

### Statistical analysis

The population for this analysis included those who reported whether or not they had received previous BoNT toxin treatment, completed the first treatment session, and completed the PNRS at baseline. The number of subjects with missing data is indicated in each table, and no values were imputed for missing data. Subjects’ pain was dichotomized into PNRS scores of 0–3 (no/mild pain) and 4–10 (moderate/severe). The PNRS was selected as the pain measure for these analyses because it was a commonly used, recommended, subject-reported measure [12], there are established cut-points [13, 14], and pain rating was independent of any other domain (in contrast to the CDIP-58 Pain and Discomfort subscale).

Two sample *t* tests and one-way analysis of variance were used to compare continuous measures between groups of two and three or more, respectively. Uncorrected Chi-square analyses were used to compare categorical measures between groups. Multinomial and logistic regression models were used to examine the effects of pain, age, and gender on employment status at study baseline and on changes in employment due to CD, respectively. Linear regression analyses assessing the relative importance of the motor component of CD (via the TWSTRS Severity Subscale) and pain (via the PNRS) to the CDIP-58 subscales utilized *R* ^2^ and Lindeman–Merenda–Gold [17] estimates, and the threshold analyses were conducted using piecewise linear regression. Linear regression models were used to examine the effects of pain, age, gender, and TWSTRS Severity on dose and the number of muscles injected. Post hoc multiple pairwise comparisons were adjusted using the step-up method of Hochberg [18]. For all analyses, a *p* value of ≤0.05 was used to reject the null hypothesis for statistical significance. All analyses were performed using R software, version 3.0.0 or greater [19]. The Lindeman–Merenda–Gold analyses were performed using the “relaimpo” package for R [20], and the piecewise linear regression analyses were performed using the “segmented” package for R [21, 22].

## Results

### Sociodemographic and clinical characteristics by baseline pain status

A total of 88 centers enrolled 1,046 subjects between January 12, 2009 and August 31, 2012. The analysis population includes 1,037 subjects who completed the first treatment session, reported whether or not they had received previous BoNT toxin treatment, and completed the PNRS at baseline. Of those, 88.9 % (922/1,037) reported pain related to CD at baseline (PNRS score >0), 70.7 % (733/1,037) rated their pain related to CD as moderate or severe at baseline (PNRS score 4–10), and 29.3 % (304/1,037) had no or mild pain (PNRS score 0–3) (Table 1). In addition, 90.6 % (863/953) of subjects reported that CD caused neck pain or discomfort prior to their study treatment. When comparing the no/mild and moderate/severe pain groups, those with no/mild pain were older (60.9 ± 14.5 vs. 56.8 ± 14.7 years, *p* < 0.0001), had higher levels of education (*p* = 0.0005), and significantly differed in predominant subtype (*p* = 0.0150). Subjects with moderate/severe pain at baseline reported significantly higher usage of analgesics, antianxiety agents, and antidepressants compared with those in the no/mild group (*p* ≤ 0.05) (Table 1). There were no significant differences between the two groups with regard to gender, race/ethnicity, BoNT-naïve status, body mass index, or time from CD diagnosis to treatment (Table 1).

**Table 1:** Sociodemographic and clinical characteristics, overall and by pain status at baseline

	Total (*N* = 1,037)	No/mild pain (*n* = 304)	Moderate/severe pain (*n* = 733)	*p* value
Age (years)				
Mean ± SD	58.0 ± 14.7	60.9 ± 14.5	56.8 ± 14.7	<0.0001
Data not available	0	0	0	
Gender				
Female	772 (74.4)	226 (74.3)	546 (74.5)	0.9608
Data not available	0	0	0	
Race/ethnicity				
White	959 (92.5)	285 (93.8)	674 (92.0)	0.2832
Non-White^a^	78 (7.5)	19 (6.3)	59 (8.0)	
Data not available	0	0	0	
BMI (kg/m^2^)				
Mean ± SD	26.6 ± 5.4	26.4 ± 5.2	26.7 ± 5.5	0.3531
Data not available	74	24	50	
Educational level				
Less than a high school diploma	41 (4.0)	9 (3.0)	32 (4.4)	0.0005
High school graduate/some college	518 (50.0)	130 (42.8)	388 (52.9)	
Associate/Bachelor’s degree	314 (30.3)	112 (36.8)	202 (27.6)	
Advanced degree (Masters, Doctoral, Professional)	147 (14.2)	50 (16.4)	97 (13.2)	
Other	17 (1.6)	3 (1.0)	14 (1.9)	
Data not available	0	0	0	
Employment status				
Retired	339 (32.7)	116 (38.2)	223 (30.4)	<0.0001
Employed full time	308 (29.7)	99 (32.6)	209 (28.5)	
Employed part time	67 (6.5)	28 (9.2)	39 (5.3)	
Disabled	123 (11.9)	15 (4.9)	108 (14.7)	
Self-employed	61 (5.9)	19 (6.2)	42 (5.7)	
Other^b^	139 (13.4)	27 (8.9)	112 (15.3)	
Data not available	0	0	0	
Work stopped due to CD^c^, *n* (%)	107 (38.5)	15 (20.5)	92 (44.9)	0.0002
Employment status affected by CD^d^				
No	327 (74.0)	134 (89.3)	193 (66.1)	<0.0001
Yes				
Different job with less responsibility/pay	28 (6.3)	5 (3.3)	23 (7.9)	
Loss of employment	4 (0.9)	0 (0.0)	4 (1.4)	
Reduced hours or responsibility	83 (18.8)	11 (7.3)	72 (24.7)	
Severity				
Mild	344 (33.2)	111 (36.5)	233 (31.8)	0.0376
Moderate	546 (52.7)	161 (53.0)	385 (52.6)	
Severe	146 (14.1)	32 (10.5)	114 (15.6)	
Data not available	1	0	1	
CD type				
Anterocollis	59 (5.7)	13 (4.3)	46 (6.3)	0.0150
Laterocollis	402 (38.8)	103 (33.9)	299 (40.8)	
Retrocollis	55 (5.3)	12 (3.9)	43 (5.9)	
Torticollis	493 (47.6)	164 (53.9)	329 (44.9)	
Other	27 (2.6)	12 (3.9)	15 (2.0)	
Data not available	1	0	1	
Age at symptom onset, years				
Mean ± SD	49.0 ± 16.7	50.1 ± 17.4	48.6 ± 16.4	0.1879
Data not available	0	0	0	
Time from CD onset to diagnosis (years)				
Mean ± SD	5.0 ± 8.1	5.6 ± 7.1	4.7 ± 8.5	0.0704
Data not available	0	0	0	
Time from CD diagnosis to treatment (years)				
Mean ± SD	1.2 ± 4.5	1.6 ± 6.0	1.0 ± 3.7	0.0840
Data not available	0	0	0	
Previously received BoNT treatment				
*n* (%)	378 (36.5)	107 (35.2)	271 (37.0)	0.5890
Data not available	0	0	0	
Concomitant medications^e^				
Vitamins and combinations	373 (36.0)	108 (35.5)	265 (36.2)	0.8482
Analgesics, miscellaneous	230 (22.2)	34 (11.2)	196 (26.7)	<0.0001
Antilipidemic agents, HMG-CoA reductase inhibitors	168 (16.2)	68 (22.4)	100 (13.6)	0.0005
Antidepressants, selective serotonin reuptake inhibitors	162 (15.6)	47 (15.5)	115 (15.7)	0.9265
β-Adrenergic blocking agents	154 (14.9)	68 (22.4)	86 (11.7)	<0.0001
Thyroid preparations	139 (13.4)	41 (13.5)	98 (13.4)	0.9598
Antianxiety agents, benzodiazepines and combinations	131 (12.6)	28 (9.2)	103 (14.1)	0.0327
Antidepressants, miscellaneous	123 (11.9)	21 (6.9)	102 (13.9)	0.0015
Proton pump inhibitors	119 (11.5)	43 (14.1)	76 (10.4)	0.0824
Data not available	0	0	0	

Data are presented as mean ± SD or *n* (%)

Pain is defined by baseline score on the PNRS: 0–3 for no/mild pain and 4–10 for moderate/severe pain

*BMI* body mass index, *BoNT* botulinum toxin, *CD* cervical dystonia, *PNRS* Pain Numeric Rating Scale

^a^Includes Asian, Black, Hispanic, Native American, and Other

^b^Includes student, unemployed, homemaker, and never employed

^c^Asked of subjects who were unemployed at study baseline (*n* = 557), but who were employed when CD symptoms began (*n* = 278)

^d^Asked of subjects who were employed at study baseline (*n* = 442); 38 subjects had never been employed

^e^Reported in >10 % of subjects

Significant differences between the groups were demonstrated when evaluating work and employment measures. Self-reported employment status differed (*p* < 0.0001) by group, with a higher percentage of those with moderate/severe pain reporting being “disabled” (14.7 vs. 4.9 %; Table 1). In addition, a multinomial regression model, in which full-time employment was the reference level, indicated that subjects with moderate/severe pain were nearly four times more likely to be disabled as an employment status [odds ratio (OR) = 3.9; 95 % confidence interval (CI) 2.2–7.2, *p* < 0.0001; Table 2] than those with no/mild pain. Age was significantly associated with differences in employment status at baseline, where the most notable shift occurred at 65 years, the standard US retirement age (Online Resource Fig. 1).

**Table 2:** Regression models of employment status, work stopped due to CD, and employment status affected by CD by pain group and gender

	Odds ratio	95 % CI	*p* value
Employment status			
Employed part time			
Moderate/severe pain	0.7	0.4–1.3	0.2881
Male	0.3	0.1–0.7	0.0060
Self-employed			
Moderate/severe pain	1.3	0.7–2.3	0.4429
Male	1.2	0.6–2.2	0.6108
Retired			
Moderate/severe pain	1.6	1.0–2.6	0.0533
Male	1.0	0.6–1.6	0.9333
Disabled			
Moderate/severe pain	3.9	2.2–7.2	<0.0001
Male	1.5	1.0–2.5	0.0799
Other^a^			
Moderate/severe pain	2.2	1.3–3.6	0.0028
Male	0.6	0.3–1.0	0.0328
Work stopped due to CD^b^			
Moderate/severe pain	2.2	1.2–4.5	0.0193
Male	1.2	0.7–2.2	0.4599
Employment status affected by CD^c^			
Moderate/severe pain	4.5	2.6–8.3	<0.0001
Male	1.1	0.6–1.8	0.8413

All values are compared with the reference of no/mild pain and female gender

Age was modeled using a cubic spline transformation with 4 *df* (three interior knots) to allow for a curvilinear relationship, and is thus not depicted in this table

*CD* cervical dystonia, *CI* confidence interval

^a^Includes student, unemployed, homemaker, and never employed

^b^For those who were unemployed at the time of study enrollment but were employed when CD symptoms began

^c^For those who were employed at the time of study enrollment

**Fig. 1:**
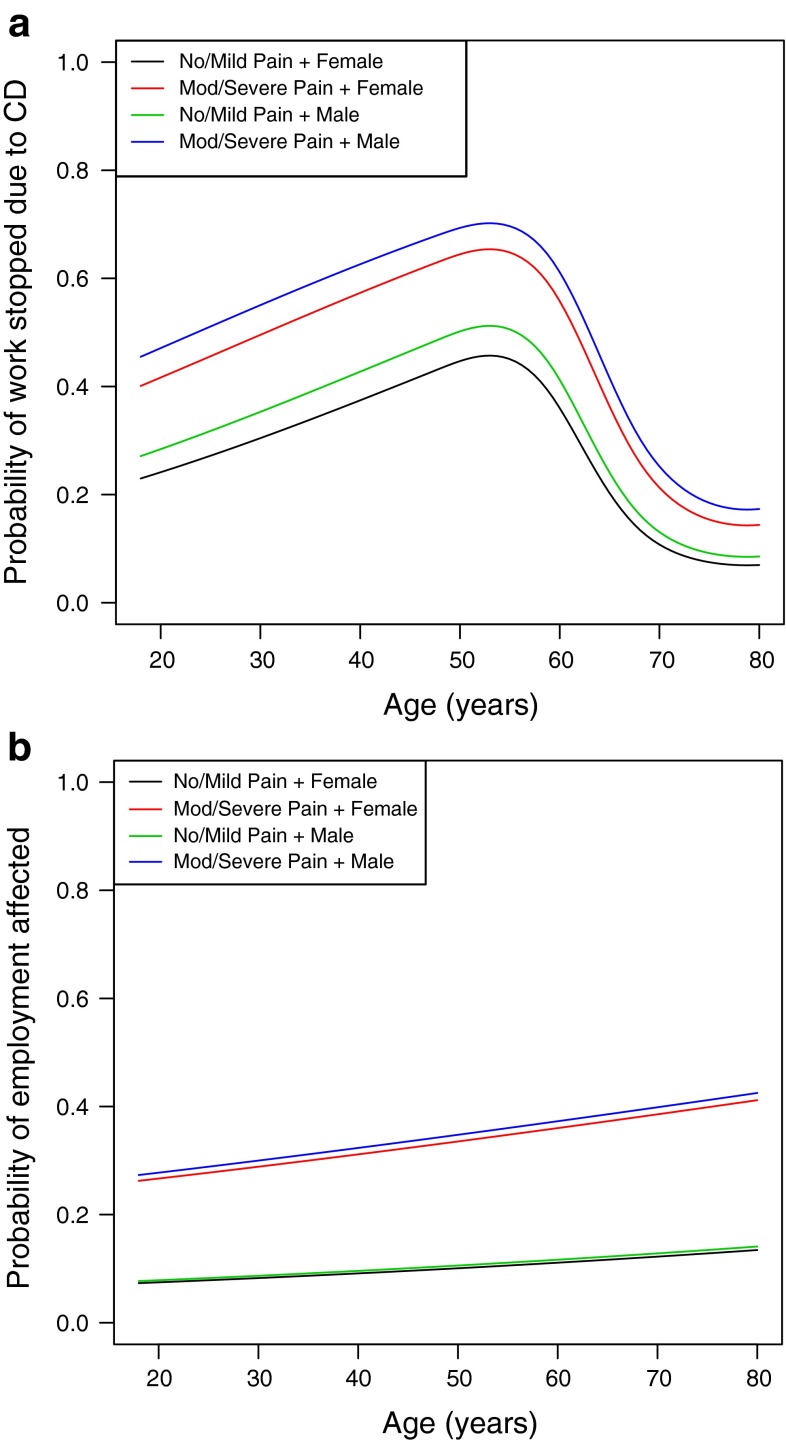
Effects of pain group, age, and gender on the **a** probability of work being stopped due to CD and **b** the probability of employment status being affected by CD. Patients had to be employed at time of CD diagnosis for these logistic regression models. *CD* cervical dystonia

In addition, those with moderate/severe pain related to CD were more likely to have reported stopping work due to CD when compared with those with no/mild pain related to CD (44.9 vs. 20.5 %, *p* = 0.0002; Table 1). Furthermore, logistic regression analysis indicated that those with moderate/severe pain were more than two times more likely to have stopped work due to CD (OR = 2.2; 95 % CI 1.2–4.5, *p* = 0.0193) than those with no/mild pain (Table 2; Fig. 1a). A general trend of an increased probability of work being stopped due to CD is seen with increasing age, until a sharp decrease beginning around age 55. For those who were employed at study baseline, a significant difference was also reported for employment status affected by CD, with a lower percentage of those with moderate/severe pain reporting no impact (66.1 vs. 89.3 %, *p* < 0.0001; Table 1). Moderate/severe pain was a significant predictor contributing to the probability that employment status was affected by CD (OR = 4.5, 95 % CI 2.6–8.3, *p* < 0.0001); there was no gender-related difference (Table 2; Fig. 1b).

### Clinical measures of pain

Baseline pain was assessed through multiple measures (mean PNRS, 5.1 ± 3.0; mean TWSTRS Pain subscale, 10.5 ± 5.1; and mean CDIP-58 Pain and Discomfort subscale score, 70.6 ± 22.8) (Table 3). Pain scales were found to correlate with one another: TWSTRS Pain subscale with the CDIP-58 Pain and Discomfort subscale (*r* = 0.63, *p* < 0.0001), TWSTRS Pain subscale with the PNRS (*r* = 0.78, *p* < 0.0001), and the CDIP-58 Pain and Discomfort subscale with the PNRS (*r* = 0.59, *p* < 0.0001). For each of these pain scales, we assessed if pain scores differed by the physician’s assessment of disease severity. There were significant differences when comparing all pain measures across the severity subgroups (mild, moderate, and severe) for the PNRS and TWSTRS Pain subscale (*p* = 0.0004 and *p* = 0.0058, respectively) (Table 3). The CD group with mild disease severity reported the lowest mean pain scores for all instruments (mean PNRS, 4.7 ± 2.9; mean TWSTRS Pain subscale, 10.0 ± 5.2; and mean CDIP-58 Pain and Discomfort subscale score, 69.7 ± 22.5) (Table 3). Furthermore, when evaluating the TWSTRS Severity and Disability subscale scores by pain status, compared with those with no/mild baseline pain, those with moderate/severe pain had significantly higher Severity (17.7 ± 5.1 vs. 16.2 ± 5.6, *p* < 0.0001) and Disability (12.7 ± 6.1 vs. 7.5 ± 5.6, *p* < 0.0001) (Table 4).

**Table 3:** Pain scores at baseline by physician-assessed severity

	Total (*N* = 1,036)^a^	Mild (*n* = 344)	Moderate (*n* = 546)	Severe (*n* = 146)	*p* value
PNRS					
*n*	1,036	344	546	146	
Mean ± SD	5.1 ± 3.0	4.7 ± 2.9	5.2 ± 3.0	5.9 ± 2.9	0.0004
Data not available	0	0	0	0	
TWSTRS Pain subscale					
*n*	1,034	344	544	146	
Mean ± SD	10.5 ± 5.1	10.0 ± 5.2	10.5 ± 5.0	11.6 ± 5.1	0.0058
Data not available	2	0	2	0	
CDIP-58 Pain and Discomfort subscale					
*n*	1,027	344	538	145	
Mean ± SD	70.6 ± 22.8	69.7 ± 22.5	70.2 ± 23.3	74.6 ± 21.3	0.0545
Data not available	9	0	8	1	

Data are presented as mean ± SD or *n* (%)

Scales range as follows: Pain Numeric Rating Scale, 0–10; TWSTRS Pain subscale, 0–20; and CDIP-58 Pain and Discomfort subscale, 0–100

*CDIP*-*58* Cervical Dystonia Impact Profile, *PNRS* Pain Numeric Rating Scale, *TWSTRS* Toronto Western Spasmodic Torticollis Rating Scale

^a^Severity data were unavailable for 1 subject

**Table 4:** TWSTRS subscale and total scores by the presence of pain at baseline, as measured on the PNRS

	Total (*N* = 1,037)	No/mild pain (*n* = 304)	Moderate/severe pain (*n* = 733)	*p* value
TWSTRS				
Severity	17.3 ± 5.3	16.2 ± 5.6	17.7 ± 5.1	<0.0001
Disability	11.1 ± 6.4	7.5 ± 5.6	12.7 ± 6.1	<0.0001
Pain	10.5 ± 5.1	5.1 ± 4.2	12.7 ± 3.5	<0.0001
Total	38.9 ± 13.1	28.7 ± 11.3	43.1 ± 11.4	<0.0001
Data not available	3	0	3	

Data are presented as mean ± SD or *n* (%)

Pain is defined by baseline score on the PNRS: 0–3 for no/mild pain and 4–10 for moderate/severe pain

Scales range as follows: Severity, 0–35; Disability, 0–30; Pain, 0–20; and Total, 0–85

*PNRS* Pain Numeric Rating Scale, *TWSTRS* Toronto Western Spasmodic Torticollis Rating Scale

### Pain and severity relationship with CD impact

Exploratory analyses were conducted to assess the interplay between pain (as measured by PNRS), the motor component of CD (as measured by TWSTRS Severity score), and CD impact (as measured by the CDIP-58 subscales). The relationship between the TWSTRS Severity and PNRS was not the same for each of the CDIP-58 subscales.

**Fig. 2:**
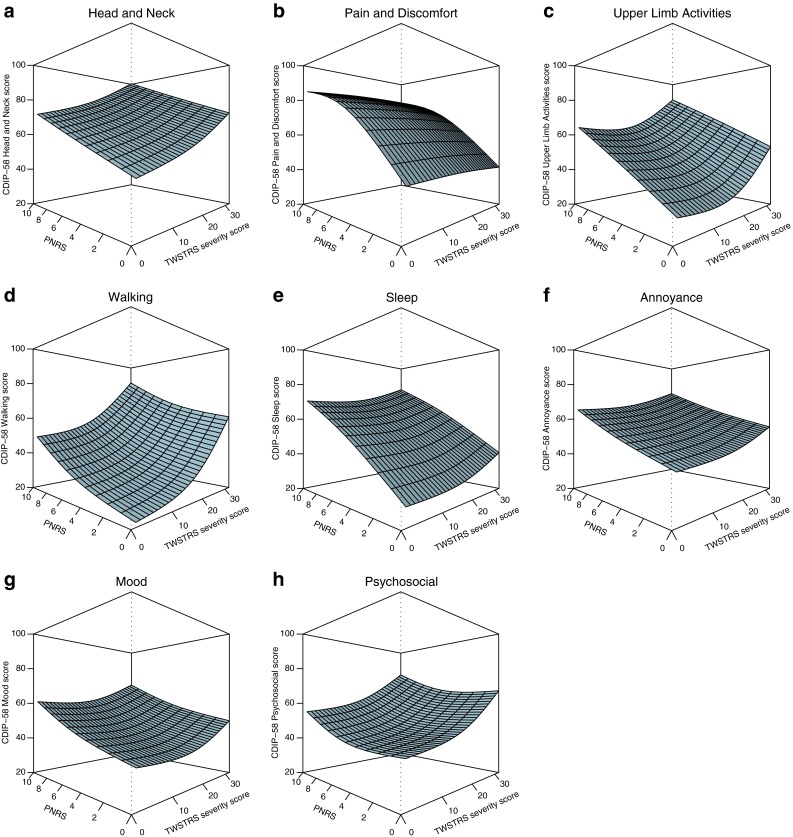
Influence of TWSTRS Severity score and PNRS score on CDIP-58 subscale scores. Lindeman–Merenda–Gold estimates and a piecewise natural cubic spline were used to generate each 3D perspective plot. *Dashed line* indicates the front of the cube for each plot; *CDIP*-*58* Cervical Dystonia Impact Profile-58, *PNRS* Pain Numeric Rating Scale, *TWSTRS* Toronto Western Spasmodic Torticollis Rating Scale

Figure 2 reflects how pain (measured by PNRS) and the motor component of CD (measured by TWSTRS Severity subscale) impact each domain of the CDIP-58 questionnaire. With the exception of the Psychosocial Functioning subscale, pain directly impacted the CDIP-58 subscales in a generally linear fashion; an increase in the pain level was associated with an increase in the CDIP-58 subscale score. While the motor component directly impacted some of the CDIP-58 subscales (Head and Neck, Walking, Annoyance, and Psychosocial Functioning subscales) in a linear fashion, it impacted the others (Upper Limb Activities, Sleep, and Mood) in a nonlinear fashion, with only a TWSTRS Severity score greater than approximately 10 demonstrating an impact. Of note, pain had a greater impact than the motor component on Pain and Discomfort, Mood, Annoyance, Sleep, Head and Neck, and Upper Limb Activities; pain and the motor component more equally impacted Walking and Psychosocial Functioning. Online Resource Table 1 provides the relative importance and *R* ^2^ values, most of which were low; thus, modeling pain and the motor component explains only a limited amount of variability in each subscale score.

Based on the findings from the 3D plots presented in Fig. 2, there appeared to be potential thresholds for when the rate of change (slope) in the CDIP-58 domain scores increases or decreases with respect to increasing PNRS and/or TWSTRS Severity scores. Piecewise regression models and subsequent evaluation of respective point estimates and CIs were conducted to determine likely threshold estimates for each CDIP-58 subscale. As suggested in the 3D plots, piecewise regression models indicated that likely thresholds were not present for every subscale. However, likely thresholds were demonstrated for PNRS in the subscale of Pain and Discomfort, with a threshold score or point estimate of 6.64 (95 % CI 5.74–7.55), and for TWSTRS Severity scores in the subscales of Upper Limb Activities, 21.83 (95 % CI 19.91–23.75); Walking, 19.05 (95 % CI 14.92–23.18); Sleep, 25.11 (95 % CI 20.67–29.55); and Mood, 25.57 (95 % CI 22.75–28.38). For all other subscales, potential thresholds were determined to be not estimable or unlikely to possible.

### OnabotulinumtoxinA treatment utilization

CD subjects with moderate/severe pain at baseline were given a significantly higher mean dose of onabotulinumtoxinA at treatment session 1 compared with those with no/mild pain (177.3 ± 82.9 vs. 158.0 ± 67.1 U, *p* < 0.0001). Similarly, subjects reporting moderate/severe pain at baseline were injected in a greater number of muscles (4.1 ± 1.4 vs. 3.7 ± 1.2, *p* < 0.0001) (Table 5).

**Table 5:** Total dose (U) and number of muscles treated at first treatment session by pain and botulinum-naïve treatment status at baseline

	No/mild pain	Moderate/severe pain	*p* value	No/mild pain + naïve	Moderate/severe pain + naïve	*p* value	No/mild pain + non-naïve	Moderate/severe pain + non-naïve	*p* value
Total dose, U (*N* = 973)^a^									
Subjects, *n*	292	681		192	419		100	262	
Mean ± SD	158.0 ± 67.1	177.3 ± 82.9	0.0001	136.6 ± 56.6	151.6 ± 64.9	0.0216	198.9 ± 67.0	218.3 ± 91.7	0.0216
Min, max	15.0, 400.0	15.0, 500.0		15.0, 346.0	15.0, 407.0		45.0, 400.0	40.0, 500.0	
Median	150.0	166.0		127.5	150.0		200.0	200.0	
Regression-adjusted mean	–	–		135.5	152.1		201.0	217.5	
Total number of muscles (*N* = 1,036)^b^									
Subjects, *n*	303	733		196	462		107	271	
Mean ± SD	3.7 ± 1.2	4.1 ± 1.4	<0.0001	3.5 ± 1.2	4.0 ± 1.3	<0.0001	4.0 ± 1.2	4.2 ± 1.4	0.7402
Min, max	1.0, 7.0	1.0, 11.0		1.0, 7.0	1.0, 10.0		1.0, 7.0	1.0, 11.0	
Median	4.0	4.0		4.0	4.0		4.0	4.0	
Regression-adjusted mean	–	–		3.6	4.0		3.9	4.2	

Pain is defined by baseline score on the PNRS: 0–3 for no/mild pain and 4–10 for moderate/severe pain

*PNRS* Pain Numeric Rating Scale

^a^Dosing information was unavailable for 64 subjects

^b^The number of muscles injected was unavailable for 1 subject

Different treatment patterns were demonstrated when comparing treatment-naïve and non-naïve groups by pain status. For both the naïve and non-naïve cohorts, subjects with moderate/severe pain received higher doses of onabotulinumtoxinA compared with subjects with no/mild pain (*p* < 0.0001 for each). When comparing within the naïve groups, a significantly higher dose of onabotulinumtoxinA at treatment session 1 was administered to those CD subjects with moderate/severe pain compared with those with no/mild pain at baseline (151.6 ± 64.9 vs. 136.6 ± 56.6 U, *p* = 0.0216) (Table 5). The number of muscles injected also was significantly greater in those with moderate/severe pain at baseline who were naïve compared with those with no/mild pain who were naïve (4.0 ± 1.3 vs. 3.5 ± 1.2, *p* < 0.0001) (Table 5). In contrast, a different pattern was seen when comparing the non-naïve groups by pain status. The mean dose was higher in the non-naïve subgroup with moderate/severe pain compared with those with no/mild pain (218.3 ± 91.7 vs. 198.9 ± 67.0 U, *p* = 0.0216) (Table 5), but the number of muscles injected was not significantly greater in those who were non-naïve and had moderate/severe pain compared with those with no/mild pain (4.2 ± 1.4 vs. 4.0 ± 1.2 U, *p* = 0.7402) (Table 5).

A linear regression model to examine the contributions of pain, age, gender, and severity (as measured via TWSTRS) on predicted onabotulinumtoxinA dose showed that subjects with moderate/severe pain received on average of 14 more units than subjects with no/mild pain (13.9 U; 95 % CI 3.1–24.7, *p* = 0.0114) (Table 6). Gender also significantly impacted dose, with males receiving nearly 15 more units than females (14.6 U; 95 % CI 3.4–25.8, *p* = 0.0109). Online Resource Fig. 2 shows that predicted dose increases with TWSTRS Severity subscale scores, and that males with moderate/severe pain received the highest doses. A similar analysis conducted for number of injected muscles showed that subjects with moderate/severe pain were injected in 0.3 more muscles than subjects with no/mild pain (0.31 muscles; 95 % CI 0.13–0.49, *p* = 0.0008) (Table 6).

**Table 6:** Dose (U) and number of muscles treated at first treatment session by pain group, age, gender, and TWSTRS Severity

	Estimate	95 % CI	*p* value
Dose, U^a^			
Moderate/severe pain	13.90	3.14, 24.66	0.0114
Age (years)	−0.48	−0.81, −0.14	0.0051
Male	14.56	3.36, 25.75	0.0109
TWSTRS Severity Score	2.33	1.41, 3.25	<0.0001
Muscles injected, *n* ^b^			
Moderate/severe pain	0.31	0.13, 0.49	0.0008
Age (years)	−0.01	−0.01, −0.00	0.0051
Male	−0.25	−0.44, −0.06	0.0089
TWSTRS Severity Score	0.03	0.01, 0.04	0.0004

All values are compared with the reference of no/mild pain and female gender

*CI* confidence interval, *TWSTRS* Toronto Western Spasmodic Torticollis Rating Scale

^a^Adjusted *R* ^2^ = 0.0502

^b^Adjusted *R* ^2^ = 0.0354

## Discussion

While the association of pain with CD has been previously described [23], the results obtained from this large cohort study clearly highlight the impact of pain upon the perceived severity, treatment paradigm, and potential effect upon work and employment.

The CD PROBE population is comparable with the CD populations from previously published literature, with the majority of subjects being female (74.4 %) and experiencing disease onset in the fifth decade of life (mean age of 49 years) [24–29]. Subjects experiencing moderate/severe pain at baseline were significantly younger than those with no/mild pain, but there was no difference in the age of symptom onset or duration of symptoms before diagnosis or treatment.

Another important finding, which should be further explored in additional analyses of CD PROBE, was the impact of CD on work and employment. A higher percentage of those with moderate/severe pain reported being disabled (though subjects could be on disability for reasons other than CD) and were more likely to have stopped work due to CD. Multinomial and logistic regression models showed that moderate/severe pain impacted employment status. Employment status and the effect of CD on employment are similar to results from other chronic pain populations [30–32]. The findings from CD PROBE indicate a significant burden to society when considering the impact of CD related to lost employment and work productivity from approximately 50 cases per million people worldwide suffering from CD [2].

The large majority of CD PROBE subjects were experiencing pain at baseline, which is consistent with other findings [24, 33, 34]. In two large prior studies of subjects with CD, the frequency of pain was 68 % [34] and 75 % [24], respectively. In this study, pain correlated with the perceived severity of CD as reported by the physician and the TWSTRS Severity and Disability subscales. These results indicate that pain correlates with disease severity, but that this relationship is complex, as it is not clear whether pain directly contributes to an increase in severity or if pain arises as a consequence of increased severity. Furthermore, these should not be considered mutually exclusive because pain may have differential impact for individual subjects. The correlations presented here explained only a limited amount of the variability in the CDIP-58 subscales, and future work could focus on identifying other contributing factors. As well, additional epidemiological studies are needed to better define the potential relationship by examining the temporal sequencing and interaction between pain and severity.

With regard to treatment, subjects with moderate/severe pain received injection in more muscles and a higher overall dose of onabotulinumtoxinA at the first injection. Moderate/severe pain, male gender, and increasing TWSTRS Severity score led to significantly higher doses at the first treatment session. However, it should be noted that these models predict only 4–5 % of the variation, so factors not identified in this analyses also influence onabotulinumtoxinA doses. When exploring the subpopulation who were toxin-naïve at baseline, those who were naïve to toxin also received a significantly lower dose at the first treatment session. However, the dose used at first injection for toxin-naïve patients is, to a degree, based on clinical judgment with regard to the potential concern of administering a new treatment, and thus doses at first treatment may not reflect an optimized treatment paradigm. Additional analyses of CD PROBE will explore how the treatment paradigm is adjusted over time and whether pain is impacted over multiple treatments.

There are several strengths related to this registry: the prospective, observational nature reflects current real-world practice, safety, and effectiveness; the large sample of CD subjects; and the use of multiple outcome measures, including those assessed by subjects and physicians. The pain scales used in this study significantly correlated with each other (though further convergent validity was not performed, as it is beyond the scope of this paper). There are also several limitations related to this registry. By design, registry studies are not blinded or randomized and lack control groups for comparison. CD PROBE did not capture the nature and pattern of pain, and it was assumed that reported neck pain was due to CD. Subgroup sample sizes differed, especially for naïve and non-naïve subgroups, which may impact the ability to interpret significance. Finally, depression status, the contribution of cervical spondylosis, and the history of injury, potentially important moderating variables, were not assessed.

## Conclusions

The results of CD PROBE more clearly elucidate the occurrence of pain and its impact upon work and treatment patterns. Most subjects report pain at baseline, and it correlates with CD severity and disability, including work and employment measures. Therefore, pain must be considered as an important factor when determining the dose and muscles injected. CD PROBE subjects with moderate/severe pain at baseline received a significantly higher mean dose and had a greater number of muscles injected upon initial treatment. Future analyses of CD PROBE will further our understanding of the treatment patterns and outcomes related to onabotulinumtoxinA therapy for this disabling condition.

## Electronic supplementary material

Below is the link to the electronic supplementary material.

Supplementary material 1 (PDF 260 kb)

## References

[1] Albanese A, Bhatia K, Bressman SB, Delong MR, Fahn S, Fung VS, Hallett M, Jankovic J, Jinnah HA, Klein C, Lang AE, Mink JW, Teller JK (2013). Phenomenology and classification of dystonia: a consensus update. Mov Disord.

[2] Steeves TD, Day L, Dykeman J, Jette N, Pringsheim T (2012). The prevalence of primary dystonia: a systematic review and meta-analysis. Mov Disord.

[3] Phukan J, Albanese A, Gasser T, Warner T (2011). Primary dystonia and dystonia-plus syndromes: clinical characteristics, diagnosis, and pathogenesis. Lancet Neurol.

[4] Molho ES, Feustel PJ, Factor SA (1998). Clinical comparison of tardive and idiopathic cervical dystonia. Mov Disord.

[5] Rondot P, Marchand MP, Dellatolas G (1991). Spasmodic torticollis—review of 220 patients. Can J Neurol Sci.

[6] Ramirez-Castaneda J, Jankovic J (2013). Long-term efficacy and safety of botulinum toxin injections in dystonia. Toxins (Basel).

[7] Hallett M, Albanese A, Dressler D, Segal KR, Simpson DM, Truong D, Jankovic J (2013). Evidence-based review and assessment of botulinum neurotoxin for the treatment of movement disorders. Toxicon.

[8] Jankovic J, Brin MF (1991). Therapeutic uses of botulinum toxin. N Engl J Med.

[9] Jankovic J, Adler CH, Charles PD, Comella C, Stacy M, Schwartz M, Sutch SM, Brin MF, Papapetropoulos S (2011). Rationale and design of a prospective study: Cervical Dystonia Patient Registry for Observation of OnaBotulinumtoxinA Efficacy (CD PROBE). BMC Neurol.

[10] Downie WW, Leatham PA, Rhind VM, Wright V, Branco JA, Anderson JA (1978). Studies with pain rating scales. Ann Rheum Dis.

[11] Cleland JA, Childs JD, Whitman JM (2008). Psychometric properties of the Neck Disability Index and Numeric Pain Rating Scale in patients with mechanical neck pain. Arch Phys Med Rehabil.

[12] Dworkin RH, Turk DC, Farrar JT, Haythornthwaite JA, Jensen MP, Katz NP, Kerns RD, Stucki G, Allen RR, Bellamy N, Carr DB, Chandler J, Cowan P, Dionne R, Galer BS, Hertz S, Jadad AR, Kramer LD, Manning DC, Martin S, McCormick CG, McDermott MP, McGrath P, Quessy S, Rappaport BA, Robbins W, Robinson JP, Rothman M, Royal MA, Simon L, Stauffer JW, Stein W, Tollett J, Wernicke J, Witter J (2005). Core outcome measures for chronic pain clinical trials: IMMPACT recommendations. Pain.

[13] Fejer R, Jordan A, Hartvigsen J (2005). Categorising the severity of neck pain: establishment of cut-points for use in clinical and epidemiological research. Pain.

[14] Zelman DC, Dukes E, Brandenburg N, Bostrom A, Gore M (2005). Identification of cut-points for mild, moderate and severe pain due to diabetic peripheral neuropathy. Pain.

[15] Cano SJ, Warner TT, Linacre JM, Bhatia KP, Thompson AJ, Fitzpatrick R, Hobart JC (2004). Capturing the true burden of dystonia on patients: the Cervical Dystonia Impact Profile (CDIP-58). Neurology.

[16] Consky E, Basinski A, Belle L, Ranawaya R, Lang AE (1990). The Toronto Western Spasmodic Torticollis Rating Scale (TWSTRS): assessment of validity and inter-rate reliability [abstract]. Neurology.

[17] Lindeman R, Merenda P, Gold R (1980). Introduction to bivariate and multivariate analysis.

[18] Hochberg Y (1988). A sharper Bonferroni procedure for multiple tests of significance. Biometrika.

[19] R Core Team (2013) R: a language and environment for statistical computing. R Foundation for Statistical Computing. http://www.R-project.org/. Accessed 25 June 2013

[20] Grömping U (2006). Relative importance for linear regression in R: the package relaimpo. J Stat Softw.

[21] Muggeo VM (2003). Estimating regression models with unknown break-points. Stat Med.

[22] Muggeo VM (2008). Segmented: an R package to fit regression models with broken-line relationships. R News.

[23] Jabbari B, Machado D (2011). Treatment of refractory pain with botulinum toxins—an evidence-based review. Pain Med.

[24] Chan J, Brin MF, Fahn S (1991). Idiopathic cervical dystonia: clinical characteristics. Mov Disord.

[25] Charles D, Brashear A, Hauser RA, Li HI, Boo LM, Brin MF, for the CD 140 Study Group (2012). Efficacy, tolerability, and immunogenicity of onabotulinumtoxinA in a randomized, double-blind, placebo-controlled trial for cervical dystonia. Clin Neuropharmacol.

[26] Comella CL, Jankovic J, Truong DD, Hanschmann A, Grafe S, on behalf of the U.S. XEOMIN Cervical Dystonia Study Group (2011). Efficacy and safety of incobotulinumtoxinA (NT 201, XEOMIN^®^, botulinum neurotoxin type A, without accessory proteins) in patients with cervical dystonia. J Neurol Sci.

[27] Lew MF, Chinnapongse R, Zhang Y, Corliss M (2010). RimabotulinumtoxinB effects on pain associated with cervical dystonia: results of placebo and comparator-controlled studies. Int J Neurosci.

[28] Chinnapongse R, Pappert EJ, Evatt M, Freeman A, Birmingham W (2010). An open-label, sequential dose-escalation, safety, and tolerability study of rimabotulinumtoxinB in subjects with cervical dystonia. Int J Neurosci.

[29] Truong D, Brodsky M, Lew M, Brashear A, Jankovic J, Molho E, Orlova O, Timerbaeva S, on behalf of the Global Dysport Cervical Dystonia Study Group (2010). Long-term efficacy and safety of botulinum toxin type A (Dysport) in cervical dystonia. Parkinsonism Relat Disord.

[30] Breivik H, Collett B, Ventafridda V, Cohen R, Gallacher D (2006). Survey of chronic pain in Europe: prevalence, impact on daily life, and treatment. Eur J Pain.

[31] Tölle T, Xu X, Sadosky AB (2006). Painful diabetic neuropathy: a cross-sectional survey of health state impairment and treatment patterns. J Diabetes Complicat.

[32] Widerström-Noga EG, Felipe-Cuervo E, Yezierski RP (2001). Chronic pain after spinal injury: interference with sleep and daily activities. Arch Phys Med Rehabil.

[33] Trosch R, Jozefczyk P, Truong D, Lew M, Adler C, LeWitt P, Singer C, Silay Y, Castagna A, Betts G, Marchese D, Comella C (2013). ANCHOR-CD (AbobotulinumtoxinA Neurotoxin: Clinical and Health economics Outcomes Registry in Cervical Dystonia): a multicenter, observational study of dysport in cervical dystonia: baseline data and cycle one efficacy data. Neurology.

[34] Jankovic J, Leder S, Warner D, Schwartz K (1991). Cervical dystonia: clinical findings and associated movement disorders. Neurology.

