# Supplementary material for: Cervical dystonia and pain: characteristics and treatment patterns from CD PROBE (Cervical Dystonia Patient Registry for Observation of OnabotulinumtoxinA Efficacy)
Source: J Neurol. 2014 Apr 22;261(7):1309–19. doi: 10.1007/s00415-014-7343-6 (PMC4098041; doi:10.1007/s00415-014-7343-6)

Supplemental data

Online Resource Table 1 Relative importance of the TWSTRS Severity score and PNRS score on the CDIP-58 subscale scores

		Joint LMG estimate	
CDIP-58 subscale	Model R^2	TWSTRS Severity	PNRS
Head and Neck	0.2754	0.3109	0.6891
Pain and Discomfort	0.3730	0.0162	0.9838
Upper Limb Activities	0.2759	0.2092	0.7908
Walking	0.2225	0.4516	0.5484
Sleep	0.2464	0.0745	0.9255
Annoyance	0.1309	0.1723	0.8277
Mood	0.1293	0.1663	0.8337
Psychosocial	0.0960	0.5235	0.4765

CDIP-58 Cervical Dystonia Impact Profile, *LMG* Lindeman-Merenda-Gold, *PNRS* Pain Numeric Rating Scale, *TWSTRS* Toronto Western Spasmodic Torticollis Rating Scale

Online Resource Fig. 1 Probabilities of employment status by pain group, age, and gender. Full time employment was the reference level in this multinomial regression model. “Other” includes student, unemployed, homemaker, and never employed.

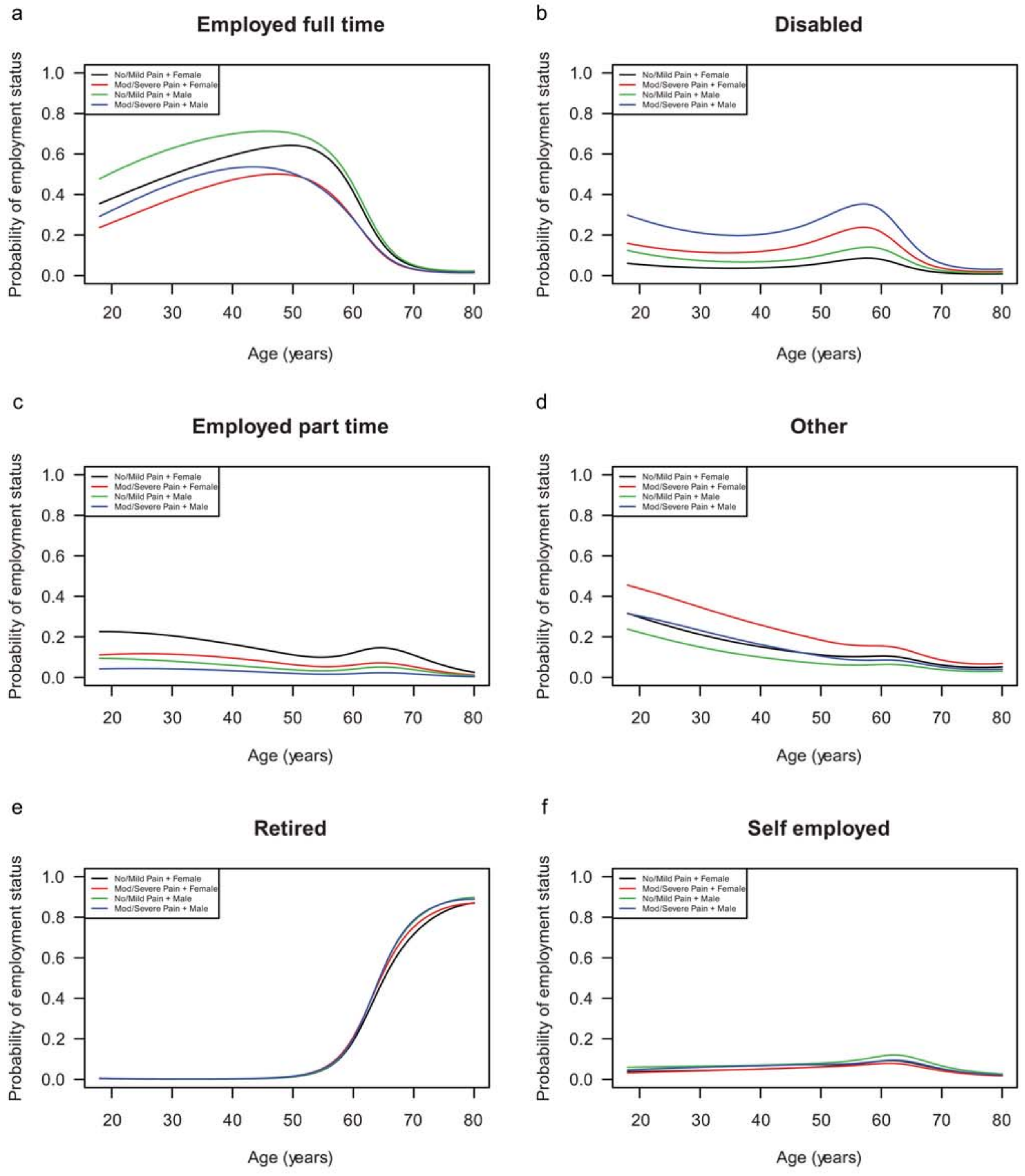

Online Resource Fig. 2 Effects of pain group, age, gender, and TWSTRS Severity subscale score on a) dose and b) number of muscles injected. Predictions are for age fixed at the observed mean of 58 years. TWSTRS Severity subscale ranges from 0–35. *TWSTRS* Toronto Western Spasmodic Torticollis Rating Scale

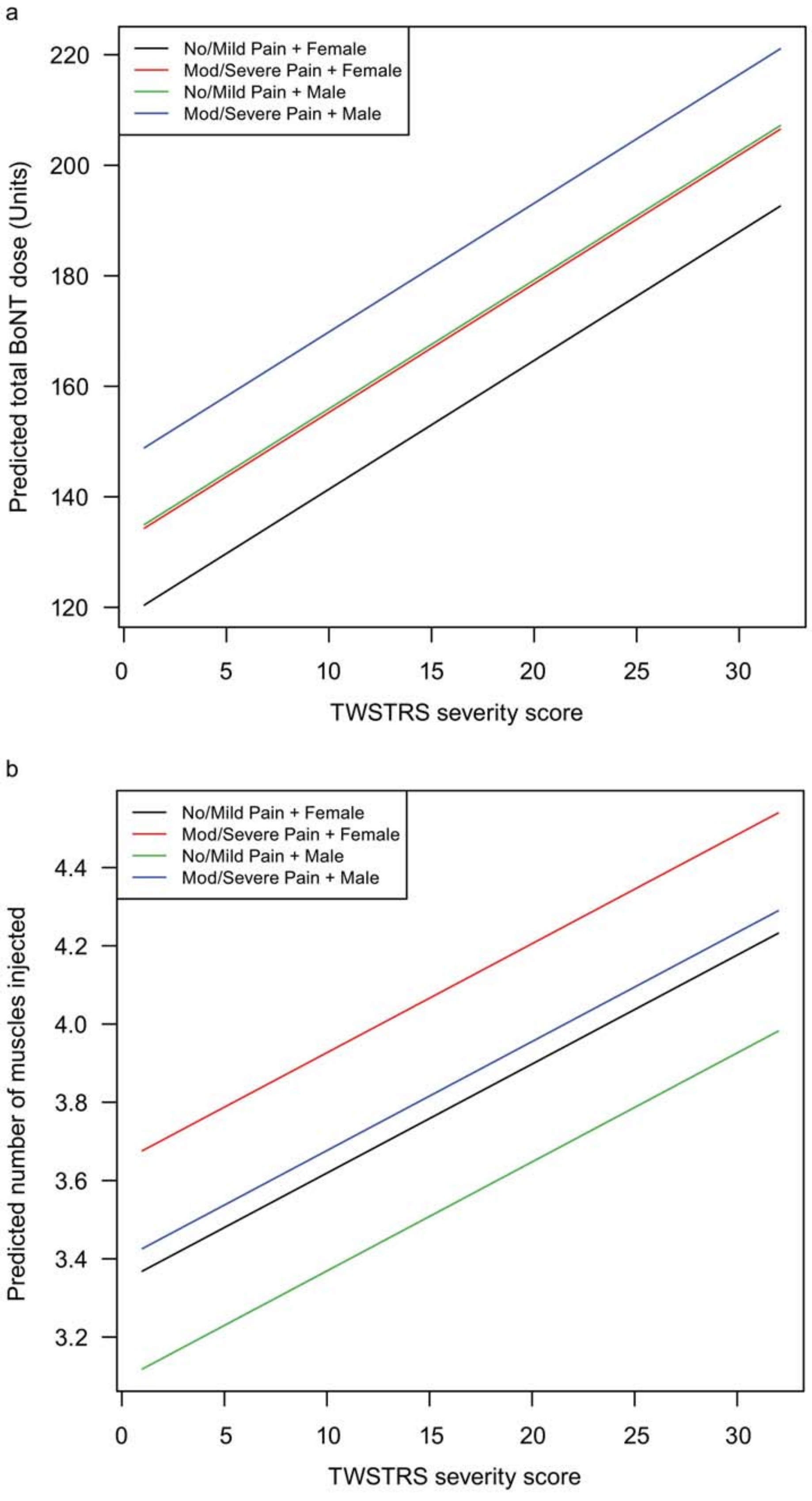

Supplement: Supplementary file 1 — Supplementary material 1 (PDF 260 kb) [file 415_2014_7343_MOESM1_ESM.pdf]
